# Does facial hair greying in chimpanzees provide a salient progressive cue of aging?

**DOI:** 10.1371/journal.pone.0235610

**Published:** 2020-07-14

**Authors:** Elizabeth Tapanes, Stephanie Anestis, Jason M. Kamilar, Brenda J. Bradley

**Affiliations:** 1 Department of Anthropology, Center for the Advanced Study of Human Paleobiology, The George Washington University, Washington, DC, United States of America; 2 Department of Anthropology, Yale University, New Haven, CT, United States of America; 3 Department of Anthropology, University of Massachusetts Amherst, Amherst, MA, United States of America; 4 Graduate Program in Organismic and Evolutionary Biology, University of Massachusetts Amherst, Amherst, MA, United States of America; Universidad de Guadalajara, MEXICO

## Abstract

The greying of human head hair is arguably the most salient marker of human aging. In wild mammal populations, greying can change with life history or environmental factors (e.g., sexual maturity in silverback gorillas). Yet, whether humans are unique in our pattern of age-related hair depigmentation is unclear. We examined the relationship between pigmentation loss in facial hair (greying) to age, population, and sex in wild and captive chimpanzees (*Pan troglodytes*). Digital facial photographs representing three chimpanzee populations (N = 145; ages 1–60 years) were scored for hair greying on a scale of one [~100% pigmented] to six [~0% pigmented]. Our data suggest that chimpanzee head and facial hair generally greys with age prior to mid-life (~30 years old), but afterwards, greying ceases to increase incrementally. Our results highlight that chimpanzee pigmentation likely exhibits substantial variation between populations, and that both 'grey' and pigmented phenotypes exist across various age classes. Thus, chimpanzee facial hair greying is unlikely a progressive indicator of age beyond mid-life, and thus facial greying in chimpanzees seems different from the pattern observed in humans. Whether this reflects neutral differences in senescence, or potential differences in selection pressures (e.g. related to conspecific communication), is unclear and worthy of more detailed examination across populations and taxa.

## Introduction

Head hair greying is one of the most important phenotypic markers for human aging, especially after mid-life [1]. An early study found that at least 50% of the aged (>50 years old) population has at least 50% grey (depigmented) scalp hair [2]. However, the age of hair greying onset and its progression throughout life are now known to vary between human populations and geographical origin [3]. Hair greying occurs from a permanent and irreversible loss of melanocytes (i.e., melanin-producing cells) in each hair follicle as humans age [4]. Therefore, melanin can no longer be deposited into hair shafts once melanocytes die. Phenotypically, the perception of grey hairs comes from an increase in depigmented hair shafts relative to the number of pigmented hair shafts (i.e., salt and pepper look in brunettes). Incremental greying is thus a progressive increase towards more depigmented hairs over time [3]. Yet, we know little about the evolutionary origin of this phenotype.

Nonetheless, we know that crypsis, signalling, and physiology all drive the evolution of coloration in wild mammals [5]. The strongest of these, crypsis, likely contributes to both drab and conspicuous coloration [6]. Color signalling plays a role in sexual communication as well as species, kin, and individual recognition, which is evinced from studies of primate faces [7]. Hair coloration may also provide thermoregulatory benefits. For example, in black fox squirrels (*Sciurus niger*), black coats often co-occur with thicker tail hairs [8]—a phenotype that potentially helps them regulate their body temperature at night. Thus, pigmentation is generally influenced by both natural and sexual selection.

In mammals, pigmentation often changes throughout an individual’s life, despite the fact that genetic variation plays an important role in producing color phenotypes [5]. These transitions may be signalled by a change in seasons (i.e., triggered by a change in photoperiod) as is the case for many arctic dwelling mammals (i.e., snowshoe hares (*Lepus americanus*), ermines (*Mustela ermina*), artic foxes (*Vulpes lagopus*)) [9]. Pelage color changes also occur alongside changes in dominance or life-history events. For example, gorilla (*Gorilla* spp.) males go through a period of greying–particularly on the lower back–and this occurs acutely at sexual maturity (~12 years) without progressing into old age [10]. Hormonal, dietary, and/or ecological factors are also thought to contribute to changes in hair pigmentation in lions (*Panthera leo*) [6, 11, 12]. Yet, several changes in pelage coloration are indeed age-related. Some pinnipeds birth melanic neonates if they give birth on islands or caves [13] and then eumelanin (black pigment) content is reduced in adult life. Despite the hypothesis that some naturally occurring mammalian populations (i.e., wild, or free-ranging captive colonies) may exhibit ‘greying’ similar to human hair [5], scientists lack empirical tests for this comparison. While evidence exists that laboratory or domestic animals grey with age [14], a recent study indicates these results may be confounded by the role acute stress plays in hair greying [15]. Instead, it remains unclear if depigmentation in pelage (i.e., hair/fur) in natural populations can progressively mark age or if it is associated with other distinct life-history or physiological changes.

Hair greying, age-related or not, is particularly interesting to examine in chimpanzees (*Pan troglodytes* ssp.) because, like humans, they are long-lived and use facial cues to discriminate faces of known individuals [16]. Thus, chimpanzee head/facial pigmentation may function as an aid to signal individual identity. Chimpanzees are also known to exhibit individual differences in general facial hair pigmentation [17], despite low population variation in coloration among the four subspecies [18]. Indeed, inter-individual variation in pigmentation has been vital for human observers' recognition of individual chimpanzees (most famously Jane Goodall’s identification and naming of the chimpanzee David Greybeard [19]). However, the extent to which hair greying is associated with and indicative of age, or other variables, remains largely untested.

In this study, we use a cross-sectional sample of chimpanzee images, to test if there is a relationship between facial hair greying with age (sex, or population). We include two subspecies of the chimpanzee, representing western (*P*. *t*. *verus*) and eastern (*P*. *t*. *schweinfurthii*) subspecies as well as captive and wild populations. We predict that if the relationship between hair greying and age is human-like, then the underlying pattern should be similar (linear, progressive, and positive as in [3]) well into old-age. Alternatively, if age-related changes in chimpanzee greying are not human-like, then we expect to find little to no association between age and hair pigmentation (in contrast to [2, 3]) and/or changes to be nonlinear.

## Materials and methods

### (A) Data collection

Digital photographs of wild and captive chimpanzee faces (N_Males_ = 94, N_Females_ = 51) of various ages (1–60 years old) were collected opportunistically from three distinct populations of two sub-species (S1 Table). Photos consist of chimpanzees in ambient light for the two wild populations (Ngogo, Kibale National Park, Uganda (*P*. *t*. *schweinfurthii*) and the South community at Taï National Park, Ivory Coast (*P*. *t*. *verus*)), and under artificial light or behind chain-link fences outside for the captive population of the New Iberia Research Center (NIRC), (*P*. *t*. *verus*). Chimpanzees were either facing the camera directly or at a slight angle (< 35°) in all photos. The years of captive chimpanzee photo collection are unknown, but wild chimpanzee photos were taken over the span of ten years between 2000–2013. Ages were categorized based on known dates of birth or estimates upon habituation based on visual cues of growth/development [20]. Twenty individuals (N_Males_ = 18, N_Females_ = 2), out of the total 145 sample, were photographed and scored at two different age-classes separated by six to thirteen years—this is representative of a small longitudinal sample within our cross-sectional design.

A large sample of independent human observers (N = 152) who were not familiar with the individual chimpanzees scored hair pigmentation phenotypes (similar to previous scorer-based studies of images in primates [21–24]). After being introduced briefly to examples of photos/individuals fitting various descriptions (S1 Fig), observers scored photos, while viewing a slideshow projected on a large screen in a darkened lecture hall. Previous work on humans verifies differences between light and brown/black hair would have minimal effects on greying [3]. Each photo was presented for no longer than five seconds, and the photo order was randomized concerning age, sex, and population. Raters scored facial/head hair greying on a scale of one to six (S2 Table) based on an assessment of normal variation in chimpanzees. We instructed raters to exclude any grey hair on the tip of the chin from their scores because juvenile chimpanzees are known to exhibit grey chin hair, and thus hair pigmentation changes in this region may be less evident. Similar scales have been previously used successfully to score differences in primate facial pigmentation [25].

#### Ethics statement

No permits were required for the described study, which complied with all relevant regulations. Long-term field sites have ongoing permits and permissions through local countries and institutions—these photos were taken opportunistically over various iterations of permits in the Ivory Coast and Uganda (not as part of the ongoing research projects).

### (B) Data analyses

#### Descriptive statistics

We created histograms to characterize the frequency of hair pigmentation across age groups for chimpanzees from mid-life onwards (individuals over 30 years old), to adequately compare the chimpanzee data to the human data after mid-life (after 50 years old) [3]. We choose 30 as mid-life for our sample since the range of ages extends between one and 60, and data indicates chimpanzees can live well into their 60s under favourable conditions [20]. The early 30s in chimpanzees has also been described as a ‘post-prime’ period [26].

For these histograms, adult chimpanzee ages were categorized into five-year intervals: from 30–34, 35–39, 40–44, 45–49 (similar to [3]). Frequency is defined as the number of individuals in a given age-class that scored a ‘2.9' or above after averaging all 152 raters’ scores, divided by the total number of individuals in that age-class. We chose ‘2.9’ as this is a conservative mid-point on our scoring scale. We left chimpanzees over age 50 out of the visualization, due to low sample size in this bracket.

#### Linear models

An analysis of covariance (ANCOVA) was run to examine the interaction effects of age and population, with sex as a co-variate, on greying. Since sample sizes were uneven in each population (Ngogo = 67, NIRC = 31, Taï = 47), we performed a type II ANCOVA using the 'car' package in R [27]. For the subset of 20 individuals for which we had longitudinal data, we used a general linear mixed model (GLMM) in R (‘lme4’ package in R) [28, 29] to examine the interaction effects of age and population, with sex as a co-variate, on greying. We included individual identity as a random effect in our GLMM in order to account for the fact that the same individuals appeared in multiple photographs. The response variable in both models was continuous (average hair greying).

We examined residual plots to be sure that the assumptions of the models were met (i.e., residuals are normally distributed). We also tested assumptions of normality across age and the average grey scores, and both datasets show they normally distributed and meet assumptions of normality independent of the other. Therefore, if age adequately predicts facial hair greying, then one can expect a positively linear relationship between both variables.

## Results

### Descriptive statistics

**Fig 1:**
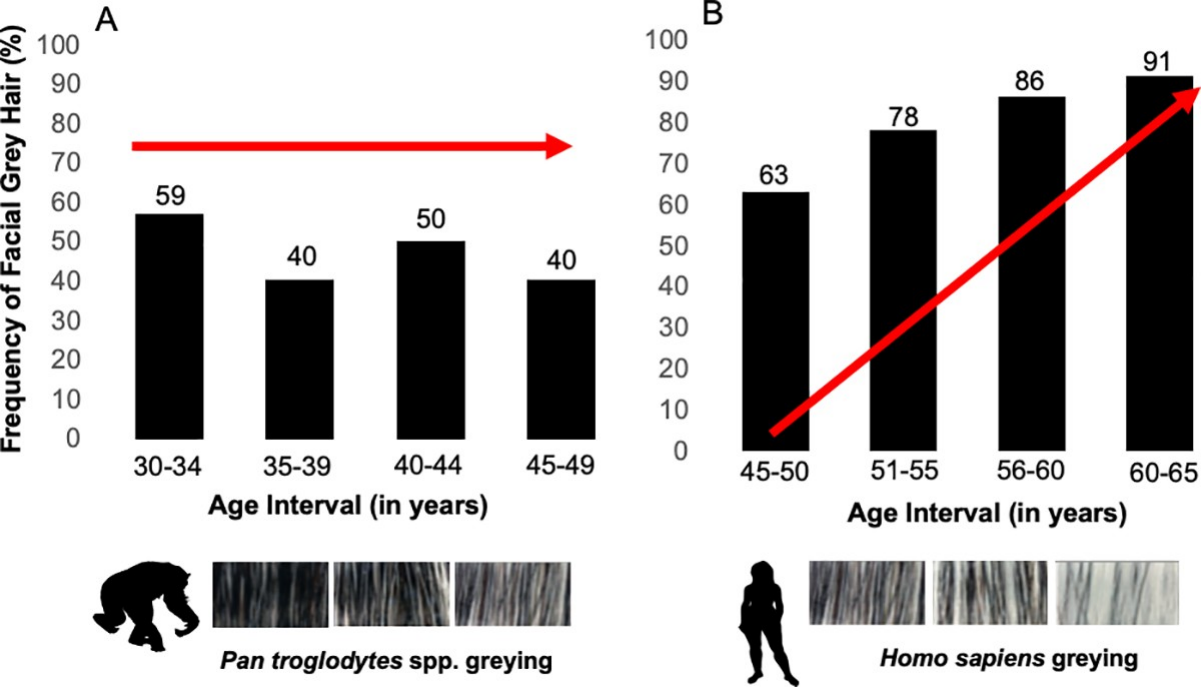
Frequency of facial hair greying after mid-life in (A) chimpanzees and (B) humans (generated from data of [3]). Greying scales below the charts (adapted from [3]) show the extent of greying observed in each species. Red arrows illustrate the direction of change in greying is (A) horizontal change, and (B) positive linear change.

The frequency of hair greying in chimpanzees is not progressive or linear after mid-life (Fig 1A). The greying pattern in chimpanzees is also unlike the pattern seen in humans (Fig 1B) where the frequency in hair greying reaches up to 91% in old age, while also exhibiting a markedly linear and progressive pattern [3]. No chimpanzee in our study scored a ‘six’ (0% pigmented hair) and only one chimpanzee approached a score of ‘five’ (~20% pigmented hair). Instead, 79% of scores in our sample fall within the range of 1–2.8 (23% scored 1–1.9, and 56% scored 2–2.8). This indicates most chimpanzees in our sample have facial hair that is <60% pigmented. This also stands in contrast to human greying, since humans frequently reach fully depigmented scalp hair. In this sample, 40% of the 20 individuals with multiple photos exhibit a 0.50 (± 0.50 SD) or less point difference in hair pigmentation, and 40% exhibit greater than one-point increase in pigmentation at different ages (S2 Fig). In the subset of individuals photographed multiple times, most changes appear trivial and exhibit no significant increases or decreases with respect to age (S2 Fig).

### Linear models

**Fig 2:**
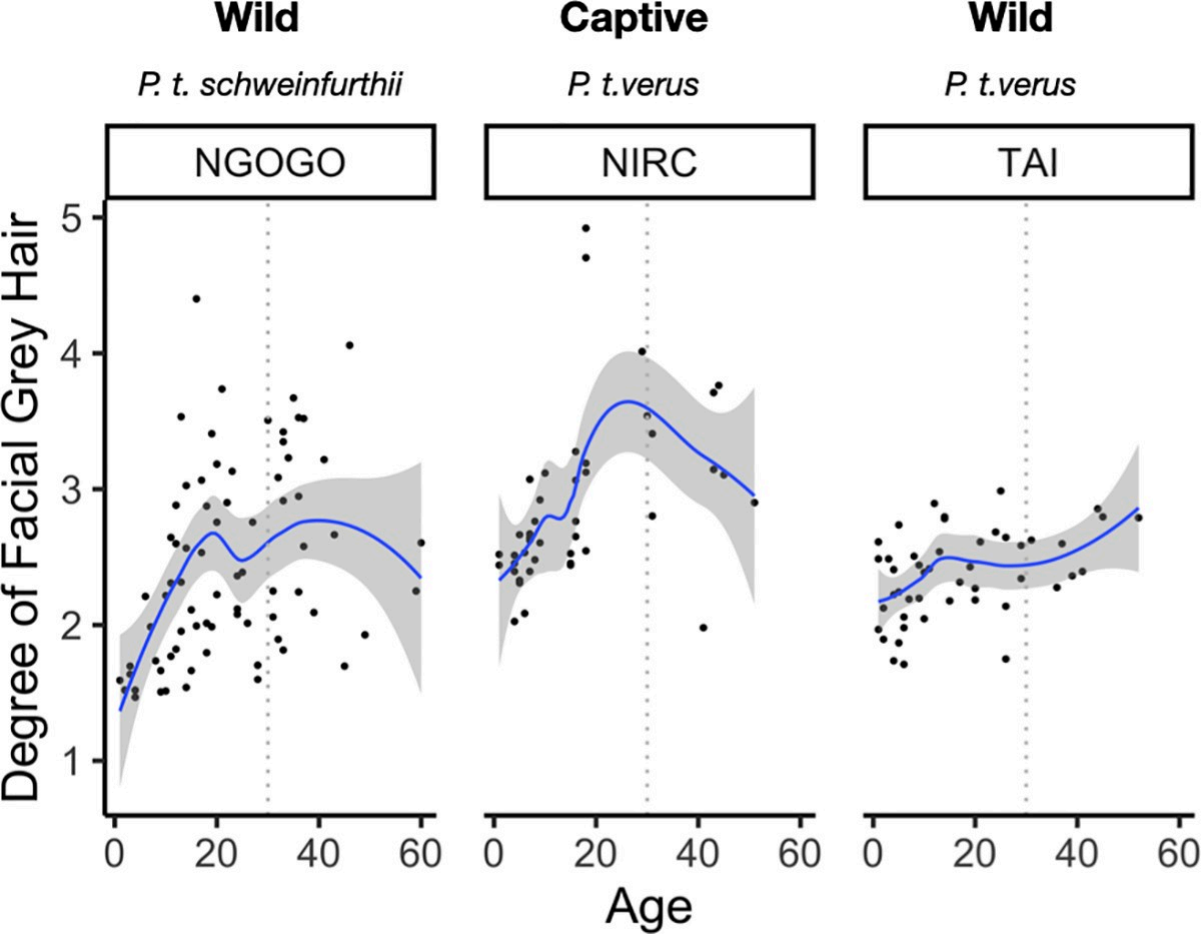
Plot of facial hair greying scores against age, fit with a smoothed line showing standard error of the regression line (i.e., grey shading). The dashed line indicates 'mid-life' for individuals in our sample (here, 30 years of age), illustrating the degree of greying does not progressively increase after this time point.

**Fig 3:**
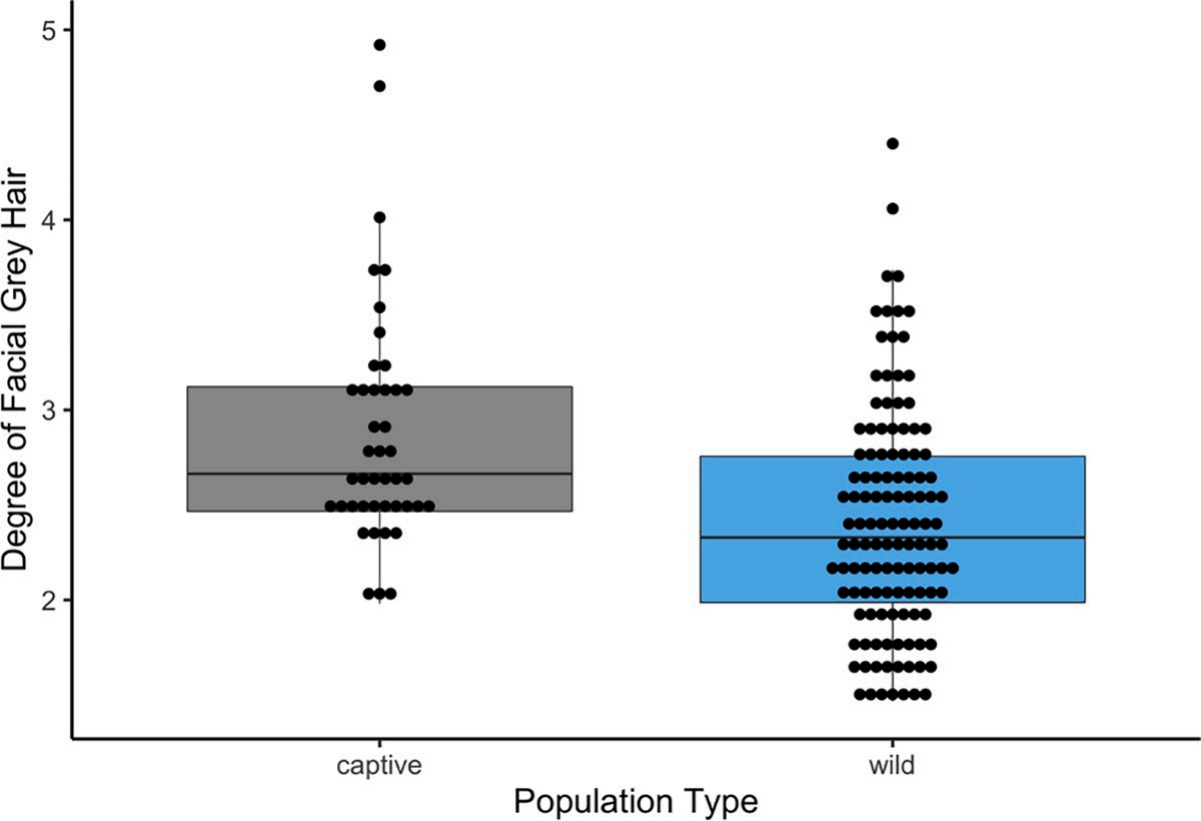
Boxplots illustrating facial hair greying variation between wild and captive chimpanzees. Black dots represent the ‘average’ grey score for distinct individuals in each population, and black lines indicate the average of the whole population.

We found significant differences in hair greying scores with age (F = 27.756, p< 0.001; Table 1) and among populations (F = 13.731, p< 0.001; Table 1) (Fig 2). From the ANCOVA and upon further visual inspection it is clear these differences indicate distinctions in greying between captive and wild chimpanzees (Fig 3), instead of differences between subspecies (S3 Fig). No statistically significant effect of sex nor an interaction between age and population were found (Table 1). However, we failed to detect a statistically significant relationship between age and hair greying scores (estimate = 0.016, p = 0.382), or any other co-variate, from the GLMM (Table 2).

**Table 1:** Summary of type II ANCOVA models examining facial hair greying against age, population, and sex in chimpanzees.

	Sum. Sq	Df	F	P value
*Hair greying*				
Age	8.762	1	27.756	**<0.0001**
Sex	0.033	1	0.748	0.7484
Population	8.669	2	13.731	**<0.0001**
Age:Population	0.538	2	0.851	0.4287
Residuals	49.877	158		

Bold values indicate statistical significance (p < 0.05).

**Table 2:** Summary of general linear mixed model (GLMM) examining facial hair greying against age and population in chimpanzees.

	Estimate	StdError	Df	T-value	P value
*Fixed Effects*					
[Intercept]	2.501	0.687	23.815	3.641	**<0.01**
Age	0.016	0.018	27.955	0.888	0.382
Sex	-0.236	0.247	20.085	-0.953	0.352
Population:NIRC	0.070	0.509	33.053	0.137	0.892
Age:PopulationNIRC	0.49	0.027	39.600	1.833	0.074
	**Variance**	**Std. Dev.**			
*Random Effects*					
Individual	0.013	0.114			
Residual	0.277	0.527			

Bold values indicate statistical significance (p < 0.05).

## Discussion

Chimpanzees faces grey with age, but unlike humans, there seems to be no marked and progressive greying after mid-life. Therefore, these pigmentation changes are not especially human-like and probably do not serve as incremental progressive cues of age for chimpanzees. Generally, facial hair greying increases with age in chimpanzees between the ages of 1 and 29 (Fig 2). In this respect, chimpanzee pigment changes are similar to those of humans. However, the patterns in chimpanzees are clearly different (Fig 1A) and show substantial inter-individual variation within each population (Fig 2). Despite finding a statistically significant effect between age and facial hair greying in our cross-sectional data models—our small sample of longitudinal photos did not detect a statistically significant effect.

Our data suggest age is a weak predictor of grey hair in chimpanzees across their lifespan and calls into question the significance of the phenotype to mark biological age in other natural mammalian populations. At best, facial greying in chimpanzees can only be used to indicate the age at which average life-expectancy has been reached (see [20]). How this compares to other naturally occurring mammal populations is unclear due to the lack of studies on hair greying as a marker of age in the wild.

The contrasting patterns of hair greying in chimpanzees and humans suggests that progressive facial/head hair greying as an indicator of age throughout adulthood in modern humans potentially originated after the chimpanzee/human lineages split, approximately 5–8 Mya [30, 31]. The lack of a progressive increase in grey facial hair after mid-life in chimpanzees’ suggests that either neutral factors or species-specific selective pressures (i.e., individual or kin recognition, crypsis, thermoregulation) likely underlie variation (or lack thereof) in chimpanzee hair pigmentation.

We found substantial inter-individual variation in greying, with each individual chimpanzee reaching a distinct greying ‘plateau’. Consistent with previous studies [24], this potentially reflects the maintenance of dark eumelanin-based coloration for crypsis or thermoregulation. Chimpanzees are also known to use facial features to discriminate between individuals [32, 33], and minimizing changes in overall facial pelage pigmentation may aid with identification (in addition to auditory, visual, and tactile cues [34, 35]). General differences in pigmentation as social identifiers are also hypothesized for Asian elephants (*Elephas maximus*) [36], and beluga whales (*Delphinapterus leucas*) [37]. Progressive facial greying into old age may be selected against in chimpanzees. Yet, we also lack empirical studies on chimpanzee greying in other body regions (i.e., back, limbs) to sufficiently understand the selective forces underlying hair color variation.

Nevertheless, genetic variation has a strong effect on hair coloration and non-adaptive explanations for the variation in facial hair greying in chimpanzees are also likely [17]. Although aging and pigmentation were the focus of this study, it is worth noting that the differences observed between chimpanzee populations (Fig 2) potentially indicate neutral non-adaptive changes (e.g., genetic drift, or pigmentation as a by-product of other aspects of physiology) since there are lineage-specific genetic differences between chimpanzee populations [38]. Nevertheless, pigmentation is usually highly heritable [39, 40], and lineage-specific patterns may explain some population-specific differences in facial hair greying specifically. However, interspecific variation in the degree of greying between captive and wild chimpanzees (Fig 3) may indicate that the proximate causes of greying may be related to variables such as stress, sun exposure, diet, or disease. Acute stress has proven to play an active role in hair greying in laboratory mice [15], and it is possible the distinction in propensity to grey between captive and wild populations is the result of similar physiological mechanisms. Genes, such as *IRF4*, also likely play a role in hair greying as it is tightly linked to the *MITF gene* (i.e., the master melanocyte regulator) in regulating pigmentation [41–43], and thus there are multiple candidate gene targets for future explorations of proximate mechanisms.

We should note that some of the variation between populations could also be due to confounding factors such as different ambient conditions where the individuals were photographed. For example, distinct lighting conditions in photographs are more likely between sites than within site. Thus, we have interpreted our results with some caution, and argue that results indicate all individuals likely reach at least 60% grey hair by mid-life, but do not continue to grey progressively afterward. Even if we assume there is some ‘noise’ in the data, then this indicates there is one earlier ontogenetic shift in pigmentation where relatively pigmented hair shows an evident increase in greying up to mid-life (< 29 yrs.), and a second phase (> 30 yrs.) typified by minimal ontogenetic shifts (or nonlinear change) in greying. However, there remains no linear pattern in old age as is typical of humans.

Another potential bias we considered was that human raters scoring the photos who are not familiar with chimpanzees could be prone to bias in scoring wrinkled looking individuals as greyer. However, we note that the oldest individuals in our sample did not necessarily score much higher than all other chimpanzees. Although screens and un-calibrated photos may also distort color perception, various studies have used similar methods to study facial pigmentation effectively in the past [24, 25, 44]. In addition, our cross-sectional methods are similar to those used in human studies [3]. The use of color charts and digital photography [45, 46] combined with longitudinal photographs from long-term study sites (i.e., Kibale National Park in Uganda; Gombe National Park in Tanzania) would be highly informative. Collecting hair samples from chimpanzee nests and quantifying the proportion of pigmented to unpigmented hairs might also hold promise, as well as measuring the spectral sensitivity of collected hair tufts [45, 46]. However, we note that studies of human hair greying have yet to use such quantitative standards [3].

Though humans and chimpanzees are both highly social long-lived animals, there are critical differences in longevity and life-history between them that may explain differences in facial greying. The best predictor of lifespan across vertebrates is age at sexual maturity [47], but there is variation in onset of sexual maturity between both populations of chimps and humans [48, 49]. Still, wild chimpanzee average life expectancy at birth falls within 15 to 33 years (converging with human hunter-gatherers), but some chimps live well into their 60s [20, 50]. Human life expectancy, on the other hand, falls into the 80s, with some humans living past 100 [51].

Age-related phenotypes might be especially important and salient for humans to indicate old-age, reduced fertility, and reproductive senescence. There is mixed evidence as to whether reproductive senescence in mid-life is unique to humans [52, 53]. It is worth noting that while there is some evidence to indicate there may be sex differences in greying for humans [54], we found no sex differences in pigmentation changes for chimpanzees. In chimpanzee societies, females maintain high birth rates into old-age, older females are preferred copulation partners, and fertility declines are generally tied to survivorship declines [23, 55]. For humans, a long survival period beyond fertile years and long-life expectancies are potentially tied to benefits of increasing a daughter’s fertility (i.e., the grandmother hypothesis). This means there are potential fitness benefits to humans surviving well-beyond fertile years [56, 57]. Grey head hair, or other age-related phenotypes, may serve a unique biological role for humans: an indication of reproductive senescence. Alternatively, greying may be more pronounced in humans since their lifespans are much longer, and this phenotype could also merely be a product of accumulated acute stress, as in laboratory mice [15].

Our results highlight that greying facial hair changes are not accurate indicators for age or sex in chimpanzees. Although grey facial hair might mark younger (<30 years old) versus older (30+ years old) age categories broadly, they are not a progressively salient gradual cue that marks age well into the maximum lifespan. This is not the pattern we observe in humans—that can often sport completely grey facial/scalp hair in their 80s. It is difficult, at best, to extrapolate or compare the results of this study to other natural mammalian populations—because the empirical data to do so, does not exist.

The lack of progressive greying revealed by this study may indicate selection on chimp pigmentation to maintain dark eumelanin coats, or that progressive greying may serve to indicate reproductive senescence in humans. Additional comparative and longitudinal data on pigmentation across individual lifetimes and great ape populations would be ideal, and we hope this study will prompt further consideration of the relationship between age and hair pigmentation in other natural mammalian populations.

## Supporting information

S1 Table Descriptive statistics for the three chimpanzee populations used in this study. NIRC = captive, and TAÏ/NGOGO = wild.
(PDF)

S2 Table Operational definitions of chimpanzee facial hair pigmentation scores. None of the individuals in this study scored a “6” for grey hair, and only one individual scored near to “5” (score: 4.90).
(PDF)

S1 Fig Illustrative scoring guide for facial hair greying scores (1–4), illustrated with *P*. *t*. *schweinfurtheii* (1a-4a) and *P*. *t*. *verus* (1b-4b).
(PDF)

S2 Fig Degree of facial hair greying changes across 20 individuals in the sample. (PDF)

S3 Fig Boxplots illustrating facial grey hair variation between the three populations. Black dots represent the ‘average’ grey score for distinct individuals in each population, and black lines indicate the average of the whole population. NIRC = captive, and TAÏ/NGOGO = wild.
(PDF)
