# Supplementary material for: Does facial hair greying in chimpanzees provide a salient progressive cue of aging?
Source: PLoS One. 2020 Jul 14;15(7):e0235610. doi: 10.1371/journal.pone.0235610 (PMC7360037; doi:10.1371/journal.pone.0235610)
Supplement: S1 Table — NIRC = captive, and TAÏ/NGOGO = wild. (PDF) [file pone.0235610.s001.pdf]

S1 Table. Descriptive statistics for the three chimpanzee populations used in this study.

Population	Subspecies	N (Total)	Females	Males
Ngogo	*schweinfurthii*	67	23	44
Tai	*verus*	47	19	28
NIRC	*verus*	31	9	22
**Totals**		145	51	94

NIRC = captive, and TAÏ/NGOGO = wild.
