# Supplementary material for: Does facial hair greying in chimpanzees provide a salient progressive cue of aging?
Source: PLoS One. 2020 Jul 14;15(7):e0235610. doi: 10.1371/journal.pone.0235610 (PMC7360037; doi:10.1371/journal.pone.0235610)
Supplement: S2 Table — None of the individuals in this study scored a “6” for grey hair, and only one individual scored near to “5” (score: 4.90). (PDF) [file pone.0235610.s002.pdf]

S2 Table. Operational definitions of chimpanzee facial hair pigmentation scores.

Pigmentation Variable	Score	Description
Hair Greying Scores	1	~100% pigmented hair (i.e., all dark hair, may show a couple white hairs below the chin)
	2	~80% pigmented hair (i.e., a few visible white hairs elsewhere on the face, excluding chin)
	3	~60% pigmented hair (i.e., at least some white hair (in a patch) visible on the face, excluding chin)
	4	~40% pigmented (i.e., white hair on the face, excluding chin, more than one patch)
	5	~20% pigmented hair (i.e., more white hair than black hair)
	6	0% pigmented hair (i.e, all white hair)

None of the individuals in this study scored a “6” for grey hair, and only one individual scored near to “5” (score: 4.90).
