# Supplementary material for: Does facial hair greying in chimpanzees provide a salient progressive cue of aging?
Source: PLoS One. 2020 Jul 14;15(7):e0235610. doi: 10.1371/journal.pone.0235610 (PMC7360037; doi:10.1371/journal.pone.0235610)

S1 Fig. Illustrative scoring guide for facial hair greying scores (1-4), illustrated with *P. t. schweinfurtheii* (1a-4a) and *P. t. verus* (1b-4b).

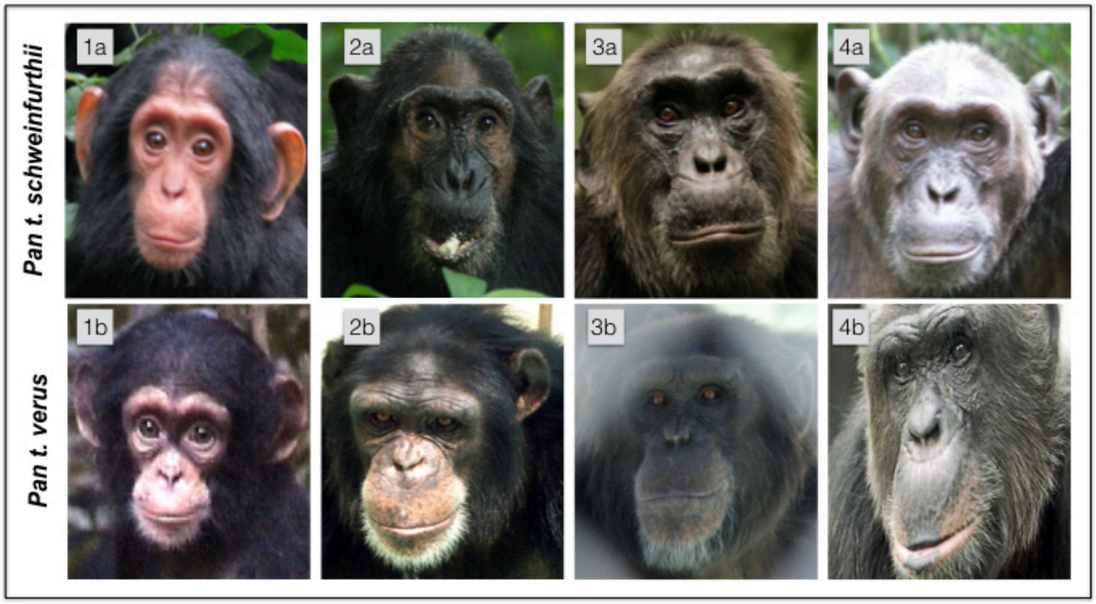

Supplement: S1 Fig — Illustrative scoring guide for facial hair greying scores (1–4), illustrated with P. t. schweinfurtheii (1a-4a) and P. t. verus (1b-4b). (PDF) [file pone.0235610.s003.pdf]
