# Supplementary figures and images for: Does facial hair greying in chimpanzees provide a salient progressive cue of aging?
Source: PLoS One. 2020 Jul 14;15(7):e0235610. doi: 10.1371/journal.pone.0235610 (PMC7360037; doi:10.1371/journal.pone.0235610)

**S2 Fig. Degree of facial hair greying changes across 20 individuals in the sample.**

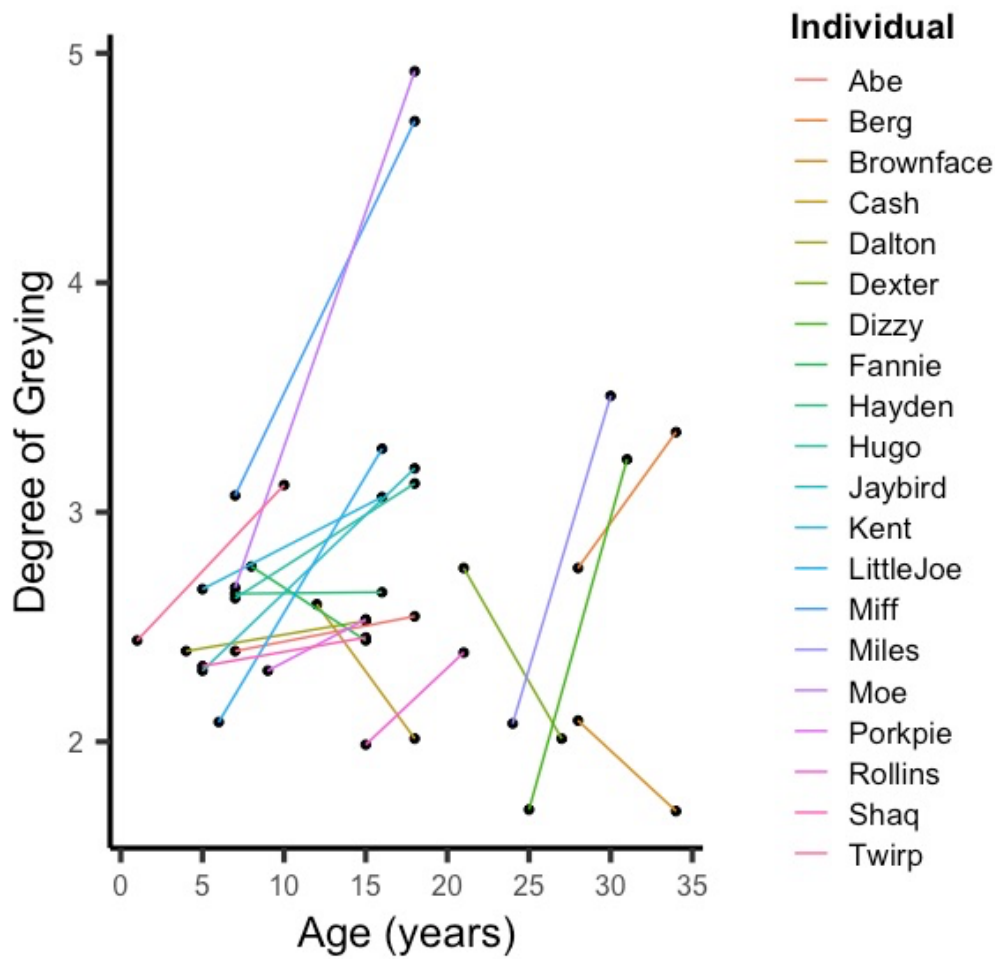

Supplement: S2 Fig — (PDF) [file pone.0235610.s004.pdf]
