# Supplementary material for: Does facial hair greying in chimpanzees provide a salient progressive cue of aging?
Source: PLoS One. 2020 Jul 14;15(7):e0235610. doi: 10.1371/journal.pone.0235610 (PMC7360037; doi:10.1371/journal.pone.0235610)
Supplement: S3 Fig — Black dots represent the ‘average’ grey score for distinct individuals in each population, and black lines indicate the average of the whole population. NIRC = captive, and TAÏ/NGOGO = wild. (PDF) [file pone.0235610.s005.pdf]

**S3 Fig. Boxplots illustrating facial grey hair variation between the three populations.**

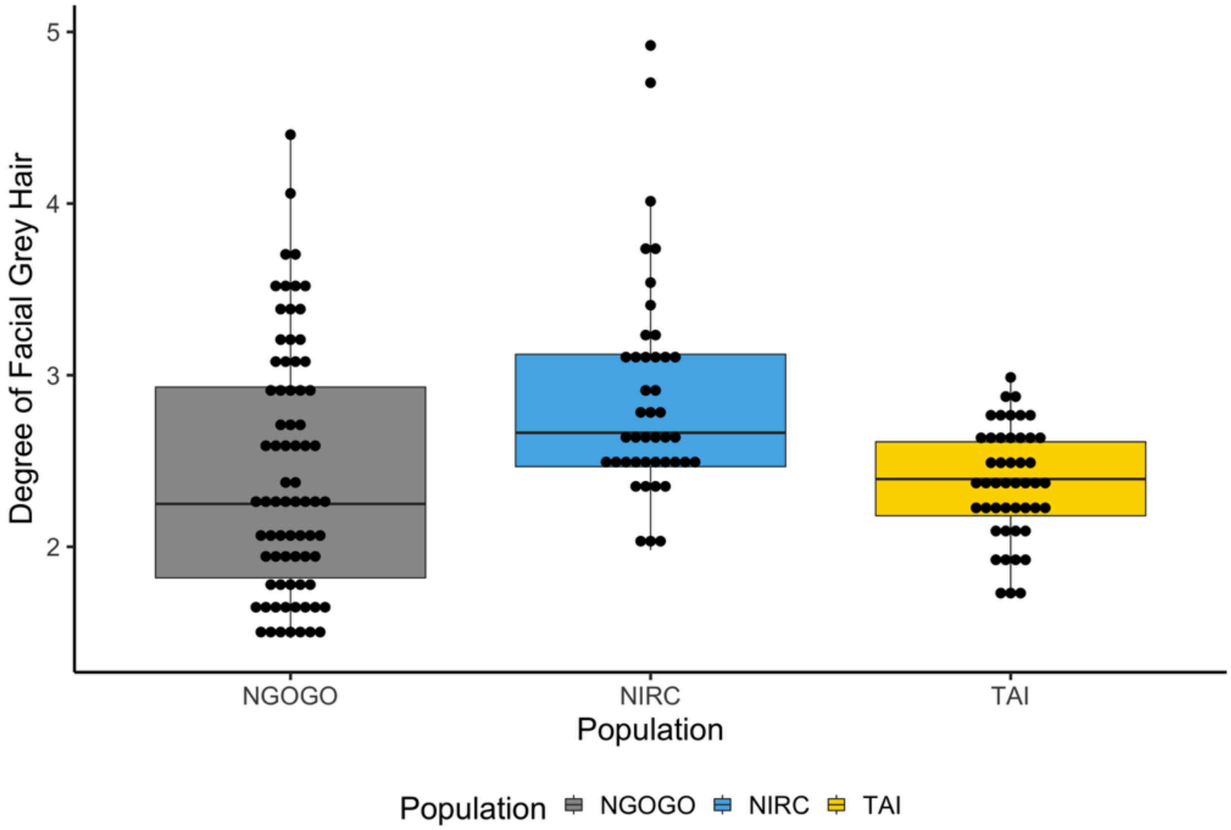


Black dots represent the 'average' grey score for distinct individuals in each population, and black lines indicate the average of the whole population. NIRC = captive, and TAÏ/NGOGO = wild.
